# Supplementary material for: Diet-Induced Glial Insulin Resistance Impairs The Clearance Of Neuronal Debris
Source: bioRxiv. 2023 Mar 10:2023.03.09.531940. Preprint. [Version 1] doi: 10.1101/2023.03.09.531940 (PMC10028983; doi:10.1101/2023.03.09.531940)
Supplement: Supplement 1 [file NIHPP2023.03.09.531940v1-supplement-1.pdf]

Figure S1. HSD does not affect glial gross morphology.

bioRxiv preprint doi: https://doi.org/10.1101/2023.03.09.531940; this version posted March 10, 2023. The copyright holder for this preprint (which was not certified by peer review) is the author/funder, who has granted bioRxiv a license to display the preprint in perpetuity. It is made available under a CC-BY 4.0 International license.

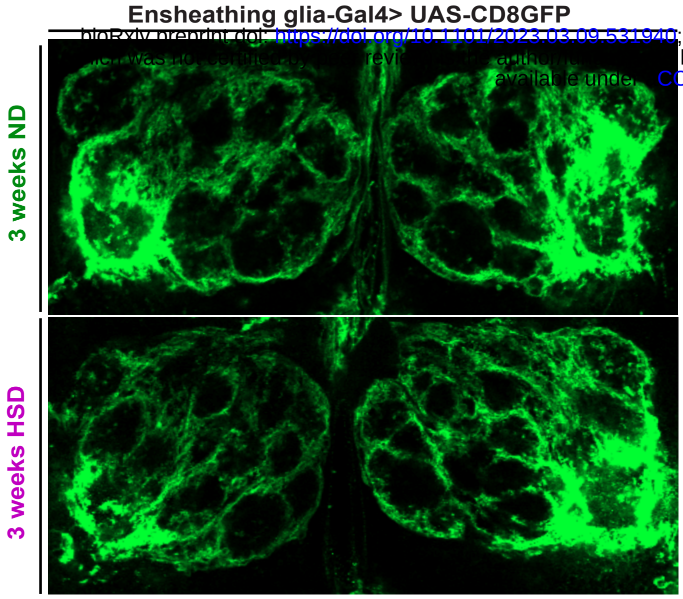

bioRxiv preprint doi: https://doi.org/10.1101/2023.03.09.531940; this version posted March 10, 2023. The copyright holder for this preprint (which was not certified by peer review) is the author/funder, who has granted bioRxiv a license to display the preprint in perpetuity. It is made available under aCC-BY 4.0 International license.

Figure S1. The antennal lobe regions of flies with ensheathing glia-driven membrane tagged GFP. No observable gross morphological defects in the HSD-fed flies.
